# Isolation and Characterization of Cetacean Cell-Derived Extracellular Vesicles

**DOI:** 10.3390/ani13213304

**Published:** 2023-10-24

**Authors:** Valentina Moccia, Cinzia Centelleghe, Ilaria Giusti, Antonella Peruffo, Vincenza Dolo, Sandro Mazzariol, Valentina Zappulli

**Affiliations:** 1 Department of Comparative Biomedicine and Food Science, University of Padua, 35020 Legnaro, Italy; valentina.moccia@phd.unipd.it (V.M.); antonella.peruffo@unipd.it (A.P.); sandro.mazzariol@unipd.it (S.M.); valentina.zappulli@unipd.it (V.Z.); 2 Department of Life, Health and Environmental Sciences, University of L’Aquila, 67100 L’Aquila, Italy; ilaria.giusti@univaq.it (I.G.); vincenza.dolo@univaq.it (V.D.)

**Keywords:** extracellular vesicles, ultracentrifugation, size exclusion chromatography, bottlenose dolphin, Cuvier’s beaked whale

## Abstract

**Simple Summary:**

Cetaceans are species of scientific interest for many reasons. First, they can be useful to assess environmental health and, second, they have peculiar features which also make them interesting for human comparative pathology. In the last decades, extracellular vesicles have been studied as important carriers in cell-to-cell communication, and many studies in human and veterinary medicine have focused on their role in pathophysiological mechanisms or as biomarker to diagnose diseases. In vitro studies are good models to explore extracellular vesicles. However, cell lines have been poorly used and investigated in these species. For these reasons, here we describe for the first time the isolation of extracellular vesicles from two cetacean cell lines established from bottlenose dolphin and Cuvier’s beaked whale. We also compare two different techniques to isolate extracellular vesicles, reporting the difference in the yield and quality of the obtained sample. This preliminary study on extracellular vesicles isolated in vitro aims to be the basis for future research to deepen our understanding on cetacean pathophysiology.

**Abstract:**

Cetaceans are of scientific interest because they are good candidates as environmental bioindicators. However, in vivo research is arduous and in vitro studies represent a rarely used valid alternative. Extracellular vesicles (EVs) are membrane-bound structures playing roles in cell-to-cell communication. Despite being a promising investigative tool in different fields of science, EVs have been poorly studied in cetaceans. To fill this gap, we describe the preliminary characterization of EVs isolated from a bottlenose dolphin and a Cuvier’s beaked whale cell line. EVs have been isolated with ultracentrifugation (UC) or size exclusion chromatography (SEC) and characterized with nanoparticle tracking analysis (NTA), Western blotting (WB), and scanning transmission electron microscopy (STEM). UC and SEC allowed the isolation of mainly small EVs (<200 nm). A higher number of particles were isolated through UC compared to SEC from both cell lines. At WB, all EVs expressed the EV-markers CD9 and integrin-β. Only EVs isolated with UC were positive for TSG101. In conclusion, we isolated for the first time EVs from a bottlenose dolphin and a Cuvier’s beaked whale cell line using two different techniques. Further studies on cell-derived EVs will be useful to deepen our knowledge on cetacean pathophysiology and health status assessment.

## 1. Introduction

Extracellular vesicles (EVs) are membrane bound nano-vesicles originating from cells and released in the extracellular space [1]. They are heterogeneous in size, ranging from 30 to 1000 nm in diameter, and, as for biogenesis, they originate mainly from endosomes or from external budding of the plasma membrane [1]. Once in the extracellular space, EVs carry RNA, proteins, lipids, and sugars from their cell of origin through body fluids and reach other cells. Therefore, they are important carriers involved in intercellular communication, having a role in the regulation of both physiological and pathological processes [2]. All these features make EVs a recent outstanding focus in many research fields, but their isolation procedure remains a critical point in all EV studies. Although different techniques and protocols have been developed to isolate EVs from different fluids and from tissues, ultracentrifugation (UC), once considered the gold standard for EV purification, is probably still the most commonly used method [3,4]. UC allows researchers to obtain a high yield of EVs, albeit with the risk of co-isolating other nanoparticles, leading to a low purity of EV sample. Other techniques, such as size exclusion chromatography (SEC) or density gradient ultracentrifugation, allow researchers to collect EVs with higher purity, despite a lower EV yield [4,5].

Most of the studies have investigated EVs in humans for their characterization and classification, to elucidate their role in physiological and pathological processes (e.g., wound healing, tumorigenesis, inflammation), and as diagnostic biomarkers or therapeutical vehicles [1,6,7]. However, EVs have also been isolated and characterized from different animal species, plants, and bacteria [8,9,10].

Among animal-derived EVs, a few preliminary studies have been performed on sea mammal-derived EVs isolated and characterized from the sera of five whales (*Balaenoptera acutorostrata*, *Balaenoptera physalus*, *Megaptera novaeangliae*, *Orcinus orca*, *Ziphius cavirostris*) and of two pinnipeds (*Halichoerus grypus* and *Phoca vitulina*), with the aim of finding possible biomarkers to assess the health status of these species [11,12]. Marine mammals, and specifically cetaceans, are considered good environmental bioindicators because of their top-level position in the trophic chain and due to their unique fat depots which tend to accumulate bio-contaminants [13]. Moreover, they have a long life span and apparently have a low incidence of cancer, making them also of interest for comparative medicine [14]. Further, studies for their conservation are strongly encouraged by national and international legal frameworks, such as the EU Biodiversity Strategy (Habitat Directive, Marine Strategy Framework Directive).

Clearly, in vivo research and sampling from free ranging cetaceans is arduous, and most of the information is obtained through the analysis of stranded animals. Therefore, in vitro research has been recently proposed as an alternative mean to better study their pathophysiological mechanisms [15].

Therefore, considering the still few in vitro studies on cetacean cell lines and the lack of in vitro studies on cetacean-derived EVs, we isolated and characterized for the first time EVs from a bottlenose dolphin (*Tursiops truncatus*) cell line and from a Cuvier’s beaked whale (*Ziphius cavirostris*) cell line using two different EV isolation methods; however, the presence of EVs in the plasma of some cetacean species has already been reported [16,17,18,19,20,21,22].

## 2. Materials and Methods

### 2.1. Cell lines

Two skin-derived fibroblast immortalized cell lines (Sea Sentinels System patent n° 102020000003248; https://www.knowledge-share.eu/en/patent/sea-sentinel-system-for-environmental-studies/ (accessed on 2 October 2023), one derived from a bottlenose dolphin and one from a Cuvier’s beaked whale, were cultured in 1× Dulbecco’s modified Eagle’s medium F12 (DMEM F12) (Thermo Fisher Scientific, Waltham, MA, USA) containing 10% fetal bovine serum (FBS; PANTM BIOTECH) and 1% penicillin/streptomycin (10 IU/mL and 10 μg/mL respectively; Corning) [16]. Cell lines were regularly tested and confirmed to be mycoplasma-free (Mycoalert Mycoplasma Detection Kit, LONZA, Basel, Switzerland).

### 2.2. Isolation of Extracellular Vesicles with Ultracentrifugation (UC) and Size Exclusion Chromatography (SEC)

To isolate EVs from each cell line, two p150 petri dishes were seeded with 3 × 10^6^ cells. Then, 24 h before EV isolation, cells were washed twice with PBS and the cell culture medium was replaced with FBS-free (FBSf) medium, in a volume of 25 mL for EV isolation with UC and of 16 mL for EV isolation with SEC.

EVs were isolated from two plates with semi-confluent cells by UC or SEC, as already described [23]. Briefly, the medium from each plate was centrifuged at 300× *g* and at 2000× *g* for 10 min at 4 °C, to remove any cell/cell debris. For EV isolation through UC, the supernatant was transferred to a 39 mL ultracentrifuge tube (Quick-Seal Round Top, Polypropylene, Beckman Coulter, Brea, CA, USA) and ultracentrifuged at 100,000× *g* for 90 min at 4 °C (Optima L-90 K, Beckman Coulter). The supernatant was discarded, and the EV-enriched pellet was resuspended in 100 μL of 0.2 μm double-filtered PBS (dfPBS). To perform EV isolation with SEC, the supernatant was centrifuged with 100 kDa ultrafiltration tubes (Amicon Ultra centrifugal filters, Merck Millipore, Burlington, MA, USA), and the concentrated medium was collected and loaded in the top of qEVoriginal/70 nm columns (IZON Science, Christchurch, New Zealand). SEC was then performed according to manufacturer’s instructions and fractions #7, #8, #9, and #10 pooled and centrifuged with a 100 kDa ultrafiltration tube (Amicon Ultra centrifugal filters, Merck Millipore). Concentrated fractions were finally collected and resuspended in 100 μL of dfPBS.

### 2.3. Nanoparticle Tracking Analysis (NTA)

After EV isolation, EVs obtained with UC or SEC from both cell lines were quantified and evaluated for particle concentration and size distribution using NanoSight NS300 (Malvern). EV samples were progressively diluted in dfPBS until the correct dilution to gain reliable measurements by NTA was reached. For each sample, camera level was set at 12, and three movies of 60 s each were recorded and analyzed using the 3.4 NTA software. For particle quantification, measurements were considered reliable when within the following instrument optimal working ranges: particles per frame from 20 to 120; particle concentration between 10^6^ and 10^9^ per mL; ratio of valid particles to total particles higher or equal to 1/5. To test differences between the groups, a statistical analysis was performed with ANOVA using GraphPad Prism 8 software. The level of significance was fixed as *p* < 0.05.

### 2.4. Protein Extraction and Western Blotting (WB) Analysis

For each cell line, cell proteins were extracted from a 15-cm plate with 90% confluent cells using 2 mL of radioimmunoprecipitation assay buffer (RIPA buffer) (Thermo Fisher Scientific) supplemented with protease inhibitor (Pierce Protease Inhibitor Tablets, EDTA-free, Thermo Fisher Scientific) according to the manufacturer’s protocol. Proteins from EVs isolated with UC or SEC were resuspended in 30 µL of RIPA buffer supplemented with protease inhibitor immediately after EV isolation.

Cells and EV-derived protein concentrations were calculated using a Pierce BCA protein Assay Kit (Thermo Fisher Scientific), according to the manufacturer’s protocol.

For WB, 20 μg of proteins extracted from cells or EVs were first denatured at 70 °C for 10 min or at 95 °C for 5 min and then resolved using NuPAGE 4–12% Bis–Tris gel (Thermo Fisher Scientific) and transferred to a nitrocellulose membrane. To block nonspecific binding sites, blots were incubated for 90 min in 5% non-fat dry milk in TBS-T (TBS containing 0.05% Tween-20) at room temperature. Then, blots were incubated at 4 °C overnight with rabbit or mouse primary antibodies against human Integrin-beta 1 (1:5000; GeneTex GTX128839, Irvine, CA, USA), TSG101 (1:1000; GeneTex GTX70255), CD9 (1:200; Bio-Rad MCA694GT, Hercules, CA, USA), and calnexin (1:1000; Cell Signaling #2679, Danvers, MA, USA) diluted in TBS-T containing 1% non-fat dry milk. Then, membranes were incubated with a peroxidase-conjugated secondary antibody (1:3000; anti-Rabbit #32260 or anti-Mouse #32230, Thermo Fisher Scientific) diluted in TBS-T for 1 h at room temperature. Reactive bands were visualized using the SuperSignal West Pico PLUS Chemiluminescent Substrate detection kit (Thermo Fisher Scientific) with the iBright instrument (Thermo Fisher Scientific).

### 2.5. Scanning Transmission Electron Microscopy

Scanning transmission electron microscopy (STEM) was performed on isolated EVs, resuspended in dfPBS, to analyze their ultrastructural morphology. According to the proper dilution to obtain the best image quality, the samples were adsorbed onto 300 mesh carbon-coated copper grids (Electron Microscopy Sciences, Hatfield, PA, USA) for 10 min in a humidified chamber at room temperature. Vesicles on grids were then fixed in 2% glutaraldehyde (Electron Microscopy Sciences) in PBS for 10 min, then briefly rinsed in Milli-Q water and negative stained with 2% phosphotungstic acid brought to pH 7.0 with NaOH. Grids with adhered EVs were examined with a Zeiss GeminiSEM 500 equipped with a scanning transmission electron microscopy (STEM) detector (Zeiss, Germany).

## 3. Results

**Figure 1:**
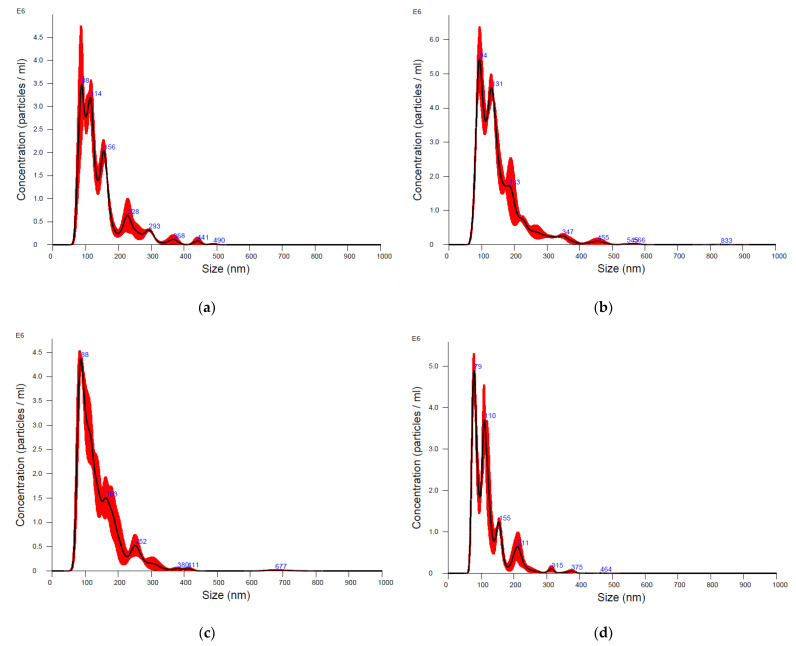
Size distribution of particles analyzed with nanoparticle tracking analysis. (**a**,**b**). Extracellular vesicles isolated with ultracentrifugation from bottlenose dolphin (**a**) and Cuvier’s beaked whale (**b**) cells. (**c**,**d**). Extracellular vesicles isolated with size exclusion chromatography from bottlenose dolphin (**c**) and Cuvier’s beaked whale (**d**) cells.

After EV purification with UC and SEC, the concentration and size of the isolated particles were measured by NTA. Results are shown in Figure 1 and Table 1.

The size distribution of particles after both the UC and SEC isolation procedure showed size ranges within the size of EVs, with the mode size of the diameter ranging from 85 to 106 nm in all samples, demonstrating the isolation of mainly small EVs (Table 1, Figure 1).

NTA showed a similar concentration of particles in EV samples isolated with UC from bottlenose dolphin and Cuvier’s beaked whale cells of 2.9 × 10^11^ +/− 2.7 × 10^10^ and 4.7 × 10^11^ +/− 2.8 × 10^9^, respectively (Table 1). The particle concentration in EV samples isolated with SEC was lower than UC samples, being 2.7 × 10^9^ +/− 1.9 × 10^8^ and 2 × 10^9^ +/− 1.4 × 10^8^ for bottlenose dolphin- and Cuvier’s beaked whale-derived EVs, respectively (*p* < 0.05) (Table 1).

**Figure 2:**
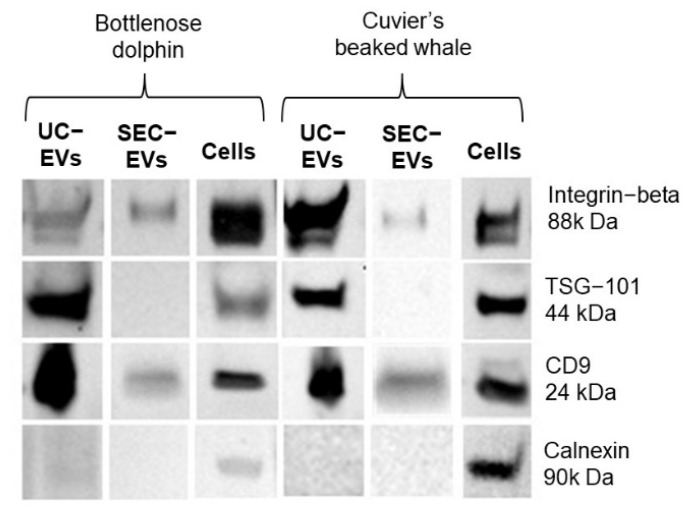
Western blot analysis performed on bottlenose dolphin and Cuvier’s beaked whale cells and extracellular vesicles samples. UC-EVs: extracellular vesicles isolated with ultracentrifugation; SEC-EVs: extracellular vesicles isolated with size exclusion chromatography.

WB was performed to characterize EVs isolated by UC and SEC from both bottlenose dolphin and Cuvier’s beaked whale cells. As a control, proteins extracted from both cell lines were used (Figure 2).

CD9 and integrin-beta 1, transmembrane proteins commonly used as EV markers, were detected in EVs isolated with UC and SEC from both cell lines and also in their cellular counterpart. TSG101, a cytosolic EV marker, was detected only in cells and EVs isolated with UC from both cell lines. Calnexin, a marker of the endoplasmic reticulum commonly used as negative control for EVs and as a positive control for cells, was not detected in EVs but was detected in both bottlenose dolphin and Cuvier’s beaked whale cells as expected (Figure 2).

**Figure 3:**
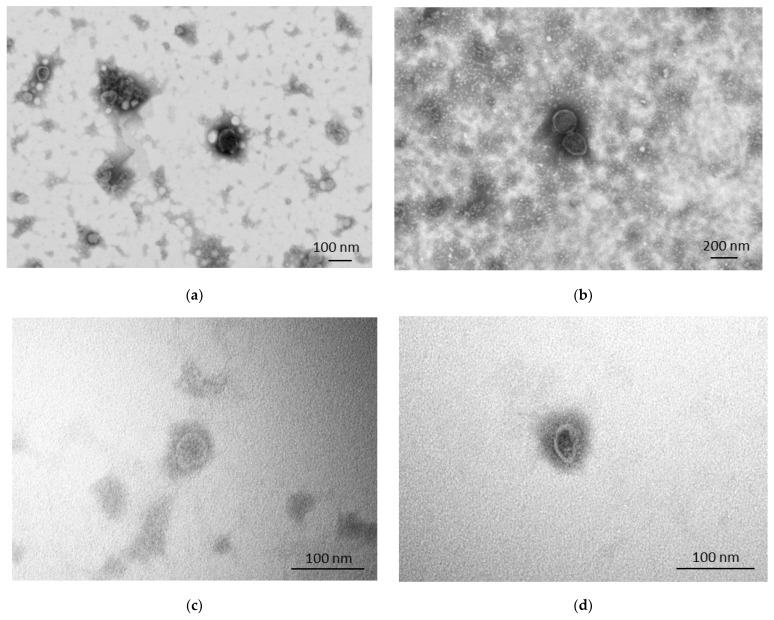
Scanning transmission electron microscopy performed on bottlenose dolphin and Cuvier’s beaked whale extracellular vesicles (EVs). (a) Bottlenose dolphin EVs isolated with ultracentrifugation (UC); (b) Cuvier’s beaked whale EVs isolated with UC; (c) bottlenose dolphin EVs isolated with size exclusion chromatography (SEC); (d) Cuvier’s beaked whale EVs isolated with SEC.

STEM further showed in the presence of intact and rounded EVs in all of our samples, as confirmed by the unbroken lipidic bilayer, that appears as a thin white filament enclosing electron dense material (Figure 3). The isolated EVs were mainly small EVs in all samples (<200 nm).

## 4. Discussion

The aim of this study was to isolate and characterize EVs from two cetaceans’ cell lines using two different isolation techniques. Similar studies have already been performed on other in vitro models [23]. However, considering the novelty of using cetacean cell lines, the fact that EVs released in vitro from cetaceans have never been described, and that EV size, concentration, and marker expression can vary according to the EV source, here we report our detailed protocol and findings. Considering the NTA, WB, and STEM results, we successfully isolated EVs from cell lines derived from bottlenose dolphin and a Cuvier’s beaked whale using both different isolation techniques. Indeed, the main differences we recorded in our EV samples were related to the isolation protocol. It is well known that UC allows researchers to isolate more particles than SEC, but it negatively affects the purity of the sample [4,5]. We saw a higher number of particles in EVs isolated with UC from both cell lines, which had a concentration of 10^11^ particles/mL compared to SEC EVs, which had a concentration of 10^9^ particles/mL. These results are partially similar to those present in another study of our research group, focused on the characterization of EVs isolated with UC and SEC from a canine mammary tumor cell line. Despite the fact that the particle concentration of UC EVs at NTA was similar, if slightly higher (7 × 10^11^ particles/mL), the SEC EV concentration from canine mammary tumor cells was higher (10^10^ particles/mL) than the concentration SEC EVs from cetaceans (ref). This might be explained by the fact that cancer cells have been demonstrated to generally shed more EVs [23].

In all our samples, particles mainly measured between 50–800 nm in diameter, a size included in the recognized size range of EVs, where EVs smaller than 200 nm of diameter are classified as small EVs and particles larger than 200 nm are classified as large EVs [24]. Considering this classification, we mainly isolated small EVs, accordingly with the used purification procedures.

Considering the protein expression, both EVs isolated with UC or SEC from bottlenose dolphin or Cuvier’s beaked whale cells expressed the EV membrane markers CD9 and integrin-beta, and all the EV samples were negative to the negative control calnexin. The absence of this protein in the endoplasmic reticulum in our samples, which was expressed instead in cells, implies the absence of a relevant quantity of cell debris. TSG101, a cytosolic marker of EVs, was only expressed on EVs isolated with UC. It is commonly recognized that UC allows researchers to collect more EVs compared to SEC and, each isolation technique can purify different subtypes of EVs, which might express different EV markers and have different biological functions [25,26]. As such, the absence of expression of TSG101 in our SEC-derived EVs might be explained by a lower quantity of TSG101 compared to UC-EVs, which might be related to the isolation of different EV subtypes with lower TSG101 expression. In relation to our previous study on canine mammary tumor cell-derived EVs, while integrin-beta and TSG101 were not tested as EV markers, CD9 was present in both UC and SEC EVs [23]. Therefore, we demonstrated that all the tested EV markers are also conserved and expressed in cetacean-derived EVs. Finally, through STEM, we clearly showed the presence of intact membranous particles, i.e., EVs, in all our samples.The results herein reported confirmed for the first time the presence of cetacean cell line-derived EVs that could be now considered to study marine mammals’ physiology and pathology in vitro, as they have been used for decades in human medicine. Moreover, despite the fact that the presence of EVs in the plasma of some cetacean species has already been reported, they had never been described in the bottlenose dolphin [11,12]. In the study of Magnadòttir and co-authors, EVs were isolated with UC from the plasma of minke whale, fin whale, humpback whale, Cuvier’s beaked whale, and orca. Since four different species were included and EVs were isolated from a different biofluid, some aspects, like particle quantification at NTA, cannot be compared with our study. However, the authors interestingly found the presence of other EV markers at WB (CD63 and flotillin-1), also showing the conservation of additional EV markers among different species [12].

Studying EVs in vivo is arduous due to their high complexity and heterogeneity. However, EVs isolated from tissues ex vivo have the advantage of coming from different cell types and, therefore, represent the tissue’s in vivo situation. However, until now, we had little information on tissue-derived EVs because of the tissue complexity and possible cell rupture that can contaminate the final EV sample [27]. EVs isolated from body fluids (e.g., blood, urine) have been investigated much more, but they are less representative than those derived from tissues [27]. Single-cell EV isolation and specific RNA sequencing analyses that allow researchers to identify the cell origin of EVs in body fluids are still pioneering and not yet standardized [28]. Therefore, despite their minor complexity and all the limits of in vitro systems, EVs released in vitro can still be useful tools to gain information on specific cell types otherwise difficult to obtain in vivo.

Both the species included in our study are relevant for conservation policies and scientific interests, but they are difficult to investigate in field conditions. Bottlenose dolphins are top predators, living in coastal environments and feeding on commercial species or interacting with fisheries and many other human related activities. For these reasons and because they are also included in Annex II of the Habitat Directive (EU Directive 92/43/CEE) they could be considered good candidates as a sentinel species to monitor both environmental and human health, taking advantage of the bioaccumulation phenomena [29,30,31]. Additionally, information gained from these species could feed the information on several descriptors of the Marine Strategy Framework Directive (EC/2008/56), including those related to the effects of contaminants (descriptor 8), marine litter (descriptor 10), and underwater noise (descriptor 11). Finally, it should also be noted that several infectious diseases of terrestrial origins and with zoonotic potential have been recently reported in *Tursiops truncatus* caused by bacterial (e.g., *Listeria monocytogenes* and *Salmonella* spp.) or protozoal (e.g., *Toxoplasma gondii* or *Giardia* spp.) agents [32,33,34,35]. Also, cetaceans could be relevant hosts for infectious diseases during spill-over events, like those which occurred in the evolution of Cetacean morbillivirus and, more recently, for a highly pathogenic influenza virus [36,37]. On the other hand, Cuvier’s beaked whales have developed efficient adaptations to deep waters and a hypoxic environment, and could be considered an interesting animal model for extreme conditions [38,39,40]. Additionally, they are very sensitive to underwater noise, causing mass strandings, in particular to specific impulsive sounds, such as mid-frequency military sonars [41,42]. It should finally be stressed that cetaceans, in general, also have a very low incidence of cancer despite their long-life span and are frequently exposed to several chemical substances often deemed to be cancerogenic, being, therefore, of interest in comparative oncology studies [43,44,45].

As described above, the EV isolation techniques included in this study have some limitations mainly related to the lower purity of the EV sample obtained by UC and to the lower yield of the EV isolated by SEC. Both isolation methods are sized-based and apply centrifugal forces with or without a gradient. Additional systems for EV isolation, such as immune-based methods could also be applied for comparison. In future studies, it would also be interesting to assess the functional effect of cetacean cell-derived EVs, investigating cellular uptake and response to external stimuli, which were not included in this preliminary investigation.

## 5. Conclusions

In this preliminary EV isolation and characterization study, we demonstrated for the first time that bottlenose dolphin and Cuvier’s beaked whale cells can release EVs in vitro, like other mammalian cells. We believe that in vitro and EV study can become an additional tool to deepen our knowledge on these threatened and physiologically unique species. Future studies will aim to expand the use of EVs in cetaceans’ cell lines to investigate their role as markers after exposure to several natural and artificial conditions, such as underwater pressure, diseases, or chemical substances, also comparing them with results obtained in vitro in other species, including human beings.

## Figures and Tables

**Table 1:** Nanoparticle tracking analysis performed on extracellular vesicles isolated with ultracentrifugation (EV-UC) or size exclusion chromatography (EV-SEC) from bottlenose dolphin or Cuvier’s beaked whale cells.

Sample	Particle Concentration/mL +/− SD *	Particle Mean Size (nm) +/− SD
EV-UC bottlenose dolphin	2.9 × 10^11^ +/− 2.7 × 10^10^	140.8 +/− 3.1
EV-UC Cuvier’s beaked whale	4.7 × 10^11^ +/− 2.8 × 10^9^	151.9 +/− 7.3
EV-SEC bottlenose dolphin	2.7 × 10^9^ +/− 1.9 × 10^8^	144.1 +/− 9.9
EV-SEC Cuvier’s beaked whale	2 × 10^9^ +/− 1.4 × 10^8^	120.7 +/− 2.9

* SD: standard deviation.

## Data Availability

All the data are available upon request to the first author.
